# miRNAs as Biomarkers and Possible Therapeutic Strategies in Synovial Sarcoma

**DOI:** 10.3389/fphar.2022.881007

**Published:** 2022-08-08

**Authors:** Shaowei Jiang, Ying Hu, Yi Zhou, Guozheng Tang, Wenxu Cui, Xinyi Wang, Bangjie Chen, Zuhong Hu, Bing Xu

**Affiliations:** ^1^ Department of Orthopedics, The First Affiliated Hospital of Anhui Medical University, Hefei, China; ^2^ Inflammation and Immune Mediated Diseases Laboratory of Anhui Province, Hefei, China; ^3^ School of Pharmacy, Anhui Medical University, Hefei, China; ^4^ The First Clinical Medical College of Anhui Medical University, Hefei, China; ^5^ Department of Orthopedics, Lu’an People’s Hospital, Lu’an, China

**Keywords:** micro RNA, synovial sarcoma, immunotherapy, engineering exogenesis, CRISPR/Cas9

## Abstract

Synovial sarcoma (SS) is an epithelial-differentiated malignant stromal tumor that has the highest incidence in young people and can occur almost anywhere in the body. Many noncoding RNAs are involved in the occurrence, development, or pathogenesis of SS. In particular, the role of MicroRNAs (miRNAs) in SS is receiving increasing attention. MiRNA is a noncoding RNA abundant in cells and extracellular serums. Increasing evidence suggests that miRNA has played a significant role in the incidence and development of tumors in recent years, including sarcomas. Previous studies show that various sarcomas have their unique miRNA expression patterns and that various miRNA expression profiles can illustrate the classes of miRNAs that may elicit cancer-relevant activities in specific sarcoma subtypes. Furthermore, SS has been reported to have the most number of differentially expressed miRNAs, which indicated that miRNA is linked to SS. In fact, according to many publications, miRNAs have been shown to have a role in the development and appearance of SS in recent years, according to many publications. Since many studies showing that various miRNAs have a role in the development and appearance of SS in recent years have not been systematically summarized, we summarize the recent studies on the relationship between miRNA and SS in this review. For example, miR-494 promotes the development of SS via modulating cytokine gene expression. The role of miR-494-3p as a tumor suppressor is most likely linked to the CXCR4 (C-X-C chemokine receptor 4) regulator, although the exact mechanism is unknown. Our review aims to reveal in detail the potential biological value and clinical significance of miRNAs for SS and the potential clinical value brought by the association between SS and miRNAs.

## Introduction

Synovial sarcoma (SS) is a high-grade soft tissue sarcoma (STS), which arises from undifferentiated mesenchymal cells and is very rare (Serinelli et al., 2021). The age-adjusted incidence is 0.81/1,000,000 in children and 1.42/1,000,000 in adults (Gazendam et al., 2021). SS can occur in almost any tissue of the human body at any age. However, SS occurs more often in soft tissues, among which the limbs are the most common. Likewise, although SS can occur in any age group, it is most prevalent in adolescents and adults under 30 (Vlenterie et al., 2015). To date, the pathogenesis of SS has not been fully elucidated. One of the causes of SS is widely thought to be a chromosome abnormality known as T (x; 18) (P11.2; q11.2) (Aytekin et al., 2020). At present, the most urgent task for treatment is to find new targets or regulation methods.

MiRNA is short RNA molecules of 19–25 nucleotides in size. MiRNA regulates gene expression after transcription and has been implicated in the development of cancer (Gillespie et al., 2018). The expression profile of miRNA varies in cancer and normal tissues. In the SS field that our research group has been focusing on, the expression of miRNA is also different in SS patients and normal people. An investigation found that serum miR-92b-3p expression levels were significantly higher in SS patients than in healthy subjects (Uotani et al., 2017a). The mechanism by which miRNA functions in the occurrence and development of tumor is still being explored, especially in SS.

In this review, the regulatory functions and mechanisms of various miRNAs in SS are summarized. Based on this, we also discussed the potential clinical value of miRNAs in the diagnosis, treatment, and prognosis of SS, which provides new thought on the clinical application of miRNAs in SS and also a new direction for subsequent research.

## Overview of Synovial Sarcoma

Synovial sarcoma is a malignant soft tissue tumor that can develop from the surface of any serosal and causes invasive metastasis (Corey et al., 2014). It accounts for 5–10% of all STS (Sultan et al., 2009). Among children and adolescents with STS, SS is the most common nonrhabdomyosarcoma STS (NRMS-STS) (Kerouanton et al., 2014). Between 1983 and 2012, the incidence of SS increased from 0.906 to 1.548 per 1,000,000,000,000 cases (Wang et al., 2017). SS can be divided into three types according to its form: monophasic SS with spindle cell bundles, biphasic SS with different areas of spindle cells and adenoid epithelium, and poorly differentiated SS commonly including sheets of small blue round cells (Guillou et al., 2004).

SS is characterized by the translocation of ss18 gene on chromosome 18 and synovial sarcoma x (SSX) gene on chromosome X (usually SSX1 or SSX2), which is the driving factor of tumorigenesis and leads to the expression of SS18-SSX fusion protein (Guillou et al., 2004). SS18-SSX fusion protein-mediated apparent genetic disorders may be related to the expression of cancer testicular antigen (CTA) (Mitchell et al., 2021). Compared with most other cancer types, the CTA in SS is extremely high and uniform (Mitchell et al., 2021). CTA is a set of tumor-associated antigens (TAA), which is highly expressed in malignant tumors, such as melanoma, lung cancer, ovarian cancer, and sarcoma but none of the normal tissues except in testicles, embryos, and placenta (Wei et al., 2020). In addition to the specificity of CTA expression, SS has another unique immune microenvironment. SS has almost no invasive T cells and low expression levels related to antigen expression, including the component of the major tissue compatibility complex. Therefore, SS has an immunology “cool” tumor microenvidic environment (El Beaino et al., 2020).

Although the pathogenesis of SS is complicated, it is also exploring clinical diagnosis methods at the same time. In an ideal sense, the diagnosis of SS should be based on the combination of test results, including the traditional morphology, identification of the chromosomal t(X;18) translocation, and a set of immunohistochemical marks (El Beaino et al., 2017). At present, T (x; 18) analysis is an important tool for the diagnosis of SS. However, the diagnosis of SS is still defective. For example, in a few cases, patients who do not carry SS18-SSX fusion genes cannot analyze and identify through T (x; 18) (Sandberg and Bridge, 2002; Storlazzi et al., 2003; Przybyl et al., 2012). At the same time, SS’s pathological diagnosis is not feasible, because the appearance of SS is overlapped with other tumor types (El Beaino et al., 2017). Another method is to diagnose the use of immunohistochemical markers. Although existing biomarkers are useful, their specificity and sensitivity are limited (Jørgensen et al., 1994; Fisher et al., 2003; Kohashi et al., 2010).

In terms of SS treatment, several methods have been applied in clinical treatment, including extensive resection, radiation therapy, chemotherapy, and targeted therapy. Among them, resection and radiation therapy are used to treat local SS, and chemotherapy and targeted therapy can be used to treat metastatic SS (Desar et al., 2018; Guo et al., 2020). Chemical therapy is divided into neoadjuvant chemotherapy and palliative chemotherapy. Neoadjuvant chemotherapy is not commonly used but is considered to be induced therapy under certain circumstances to improve the surgical results of the limbs and chest wall high-risk sarcoma (Le Cesne et al., 2014). Palliative chemotherapy is mainly suitable for metastatic SS patients. At present, doxorubicin is a standard first-line drug for palliative chemotherapy. It is also reported that a more beneficial method is a combination of ifosfamide and doxorubicin (Desar et al., 2018). Thus far, the available treatment drugs for targeted therapy are limited, and Pazopanib is still the only targeted drug that approves for the treatment of SS (Desar et al., 2018). Generally speaking, although there are many treatment methods for SS, there remain difficulties in the treatment of advanced metastasis in the treatment, and there is no approved systematic therapy for histological or genomic characteristics.

The treatment method mentioned above does not achieve the ideal effect in the treatment of metastatic SS. Therefore, new treatment methods should be developed, and immunotherapy is one of them. Immunotherapy is to manipulate the immune response to tumors for the purpose of treatment (Esfahani et al., 2020).

The implementation of immunotherapy must consider the immunology characteristics of cancer. The immunology characteristics of SS are as follows. As a kind of genetically simple tumors, SS has a single disease-specific translocation and a limited number. In addition, SS expresses high levels of self-antigens, such as New York squamous cell carcinoma 1 (NY-ESO-1) (Lai et al., 2012; D'Angelo et al., 2018). NY-ESO-1 is a tumor-related antigen (TAA), which is expressed in approximately 70% of SS (Ramachandran et al., 2019). In addition to TAA, there are tumor-specific antigens on SS cells, such as P53, RAS, and fusion-derived neoantigens produced by the t (X; 18) (p11; q11) (Kawaguchi et al., 2012). These immunology characteristics of SS make the treatment strategy of T cell infiltration more beneficial to the treatment of SS.

Immunotherapies of SS include T cell receptor (TCR) gene therapy and cancer vaccine. TCR gene therapy is performed by isolating peripheral blood T cells and genetically modifying them under *in vitro* conditions to express TCRs that target specific cancer antigens for the use of adoptive cell therapy (ACT) (Dallos et al., 2016). Thus far, NY-ESO-1 is the most researched ACT antigen target in SS (Nakata et al., 2021). The results of a clinical trial show that 36% of SS patients receiving TCR gene therapy have achieved meaningful clinical results in the test group (Ramachandran et al., 2019). According to previous studies, NY-ESO-1 TCR gene therapy may be a good candidate for metastatic SS (Robbins et al., 2011; Robbins et al., 2015). Cancer vaccines use SYT-SSX fusion peptide, which results from characteristic SS translocation as vaccines for advanced SS patients (Kawaguchi et al., 2012). Cancer vaccine is usually combined with other immunotherapy, and clinical trials show possible effects (Kawaguchi et al., 2012). All in all, SS immunotherapy is hopeful, but it is still under development and must overcome many challenges.

Therefore, to diagnose and treat SS, new ideas are required. It is worth noting that miRNA, as a commonly used regulator for gene expression, is considered to be a potential new choice for diagnosis and treatment of local recurrence of SS or distant metastasis (Fricke et al., 2015).

## Overview of microRNA

miRNA is a small endogenous RNA that has been found first in C. elegans and can affect the posttranscriptional gene expression of it. It is found in a wide range of species from single-celled algae to humans. At the same time, miRNA has a wide tissue distribution, most of which is located in cells but also exists in a large number of extracellular serums (Hanson et al., 2009; Wang et al., 2010; Weber et al., 2010; Zubakov et al., 2010; Zen and Zhang, 2012). The process of miRNA generation is the modification of primary miRNA (Pri-miRNA) into precursor miRNA (Pre-miRNA) through complex nuclear and cytoplasmic mechanisms (Carmell and Hannon, 2004). Thus, hairpin-shaped Pre-miRNAs are transformed into mature miRNAs in response to the cytoplasmic ribonucnavase III enzyme (Ha and Kim, 2014). miRNA leads to corresponding mRNA degradation and/or translation inhibition by inducing silencing complex *via* RNA binding to the 3′ untranslated region of the target mRNA (Smolle et al., 2017).

MiRNA is “extracellular communication RNA” that plays a role in cell proliferation and differentiation. It is involved in immune response, neurodevelopment, DNA repair, apoptosis, oxidative stress, and tumorigenesis. Therefore, abnormal miRNA expression plays a role in a variety of disease processes, including cancer. Abnormal expression of specific miRNA is almost common in malignant cancer patients, which may be related to the occurrence, maintenance, or progression of malignant cancer (Calin et al., 2002; Kumar et al., 2007; Wang et al., 2013). As a tumor suppressor, the expression of specific miRNA is downregulated in the corresponding malignancy or overexpressed to play a carcinogenic role (D'Angelo et al., 2016). Specific mechanisms of miRNA abnormal expression include defective processing pathways, epigenetic modifications, or miRNA gene mutations, which lead to miRNA dysfunction in human cancer. For example, in lung cancer and acute myeloid leukemia, miR-29b plays a role through the demethylation and reactivation of relevant cancer suppressor genes (Amodio et al., 2013). Furthermore, as an effective miRNA oncogene, miR-17–92, is highly expressed in a variety of hematopoietic malignancies and a variety of solid tumor types (Olive et al., 2013). As a certainty, miRNA also functions in SS. Previous studies have shown that miR-494-3pplays a cancer suppressor role in SS through modulating CXCR4. In conclusion, miRNA, as a common gene expression regulator, is related to the occurrence and development of many cancers, including SS, and may be used to diagnose and treat local recurrence or distant metastasis of SS in the future.

## Function Role of miRNAs in Synovial Sarcoma

Abundance of evidence has demonstrated that miRNA plays a significant role in the incidence and development of tumors in recent years, including sarcomas. Previous studies have indicated that miRNA expression profiles could elucidate classes of miRNAs that may elicit tumor-relevant activities in specific sarcoma subtypes (Yu et al., 2016). Various miRNA expression patterns have been reported unique to many of the different classes of sarcomas, which could potentially be used to identify the histologic type of sarcoma (Miska, 20052005; Subramanian and Kartha, 20122012). Furthermore, relevant research results showed that in sarcomas, SS had the most number of differentially expressed miRNAs with 99 overexpressed miRNAs and 24 underexpressed miRNAs (Yu et al., 2016). This suggested the potential importance of miRNAs in SS. In addition, with a further investigation of unique miRNA expression patterns, it could be very probable to get a better understanding of the oncogenic mechanisms and future therapeutic strategies for SS (Subramanian and Kartha, 20122012). Among these miRNAs in SS, the upregulated expression of miR-17, miR-214, miR-92b-3p, and miR-9 and the downregulated expression of miR-494-3p have been reported in terms of previous publications. The regulation expression in SS and the location on chromosome of miRNA are listed in Table 1. In this section, we will have a conclusion on the effects of these miRNAs mentioned above in the occurrence and development of SS.

**TABLE 1:** The Regulation Expression in SS and the Location on Chromosome of MiRNA.

miRNA	Chromosome location	Expression in SS	Reference
miR-494-3p	14q32.31	downregulation	miRBase
miR-17	13q31.3	upregulation	Yu et al. (2022)
miR-214	1q24.3	upregulation	Weber (2005), Scott et al. (2012)
miR-92b-3p	1q22	upregulation	miRBase
miR-9	1q22 (miR-9-1) 5q14.3 (miR-9-2) 15q26.1 (miR-9-3)	upregulation	Roese-Koerner et al. (2016)

### miR-494-3p

miR-494-3p is a multifunctional miRNA located on human chromosome 14 and is linked to many biological processes in the human body. To date, miR-494-3p has been detected in multiple human tissues, such as the lung tissue, prostate tissue, breast tissue, and retina tissue. More and more research studies have shown that several pieces of research are related to miR-494-3p accumulated in the field of cancer, and there are associations that have even been identified by some experimental research studies between miR-494-3p and certain malignancies. For example, miR-494-3p has been reported to be downgraded in prostate cancer (Ambs et al., 2008), lung cancer (Ohdaira et al., 2012), and head and neck squamous cell carcinoma (Kumar et al., 2007) and to be upregulated in retinoblastoma (Zhao et al., 2009). It should be noted that miR-494 has been demonstrated by previous publications to be downregulated or correlated with poor prognosis in cancers, and the overexpression of miR-494 could inhibit the proliferation, migration, and invasion in breast cancer (Zhan et al., 2017), ovarian cancer (Han et al., 2016; Yuan et al., 2016), gastric cancer (Zhao et al., 2016), and osteosarcoma (Zhi et al., 2016). Moreover, because miR-494-3p is downregulated in SS, miR-494-3p may have similar effects in SS.

In addition, it is worth noting that some scientific studies, including many of those that examine these malignant tumors mentioned above associated with miR-494-3p, have indicated that there is a biological association between miR-494-3p and a certain chemokine receptor—C-X-C chemokine receptor 4 (CXCR4)—in different malignant tumors. As a seven-transmembrane G protein-coupled chemokine receptor, CXCR4, which plays a role in cell motility, invasion, and angiogenesis in tumor cells, has also been found to be a prognostic marker in various types of cancer and to be upregulated in cancer metastasis, thereby implying the importance of CXCR4 to the development of tumors (Furusato et al., 2010). Furthermore, in many kinds of cancers, the C-X-C motif chemokine ligand 12 (CXCL12)/CXCR4 axis plays a role in tumor formation and metastatic dissemination. A series of proofs by several researchers showed the presence of influence of the CXCR4-SDF-1 (CXCL12) axis on tumors: many kinds of tumors, including breast cancer, ovarian cancer, prostate cancer, rhabdomyosarcoma, and neuroblastoma metastasize to the bones through the bloodstream in a CXCL12-dependent manner (Geminder et al., 2001; Müller et al., 2001; Libura et al., 2002; Dontu et al., 2003; Hall and Korach, 2003; Jankowski et al., 2003; Sun et al., 2003; Porcile et al., 2004).

In prostate cancer cells, CXCR4 has previously been characterized as a target gene of miR-494-3p. Some researchers who analyze the potential role and mechanism of miR-494-3p in regulating CXCR4 posttranscriptionally in human prostate cancer cell lines. Then, the results showed that the overexpression of miR-494-3p might play a crucial role in posttranscriptionally regulating CXCR4 in human prostate cancer cells (Shen et al., 2014). Meanwhile, miR-494-3 has been implicated as a regulator of CXCR4 not only in prostate cancer but also in many other tumors, including SS, according to previous bioinformatics and biological evidence. It indicated that miR-494.3p may be related to the progression of SS through targeting CXCR4.

A previous study by a research team has shown that CXCR4 plays a facilitating role in the migration and invasion of SS cells and that miR-494-3p functions in SS through the CXCR4 as a potential target gene (Pazzaglia et al., 2019).

Because earlier research showed that high levels of CXCR4 expression are linked to a poor prognosis and a high incidence of metastasis in bone and STS (Kim et al., 2011; Li et al., 2017), the results of the research team mentioned in the preceding paragraph (Pazzaglia et al., 2019) suggested that CXCR4 plays an important role in cancer cell movement between the main and metastatic sites in SS. Therefore, the research team mentioned in the preceding paragraph examined CXCR4 mRNA levels in a series of monophasic localized and metastatic SS specimens and discovered considerably increased expression of the CXCR4 gene in the tumor compared to normal tissue, confirming that CXCR4 promotes the incidence and progression of SS (Pazzaglia et al., 2019). In their experiments, which included analyzing CXCR4 and miR-494-3p expression in SS surgical specimens as well as some vitro experiments, the data revealed a significant negative correlation between CXCR4 and miR-494-3p expression in SS surgical specimens. They also found that the CXCR4 expression is lower and the miR-494-3p expression is higher in nonmetastatic patients than in metastatic ones. Therefore, low expression of miRNA-494-3p signals the increased expression of CXCR4, thus promoting the development of SS. The eventual result from the research team revealed that ectopic expression of miR-494-3p inhibited SS cell proliferation and migration through modulating CXCR4 (Pazzaglia et al., 2019).

**FIGURE 1:**
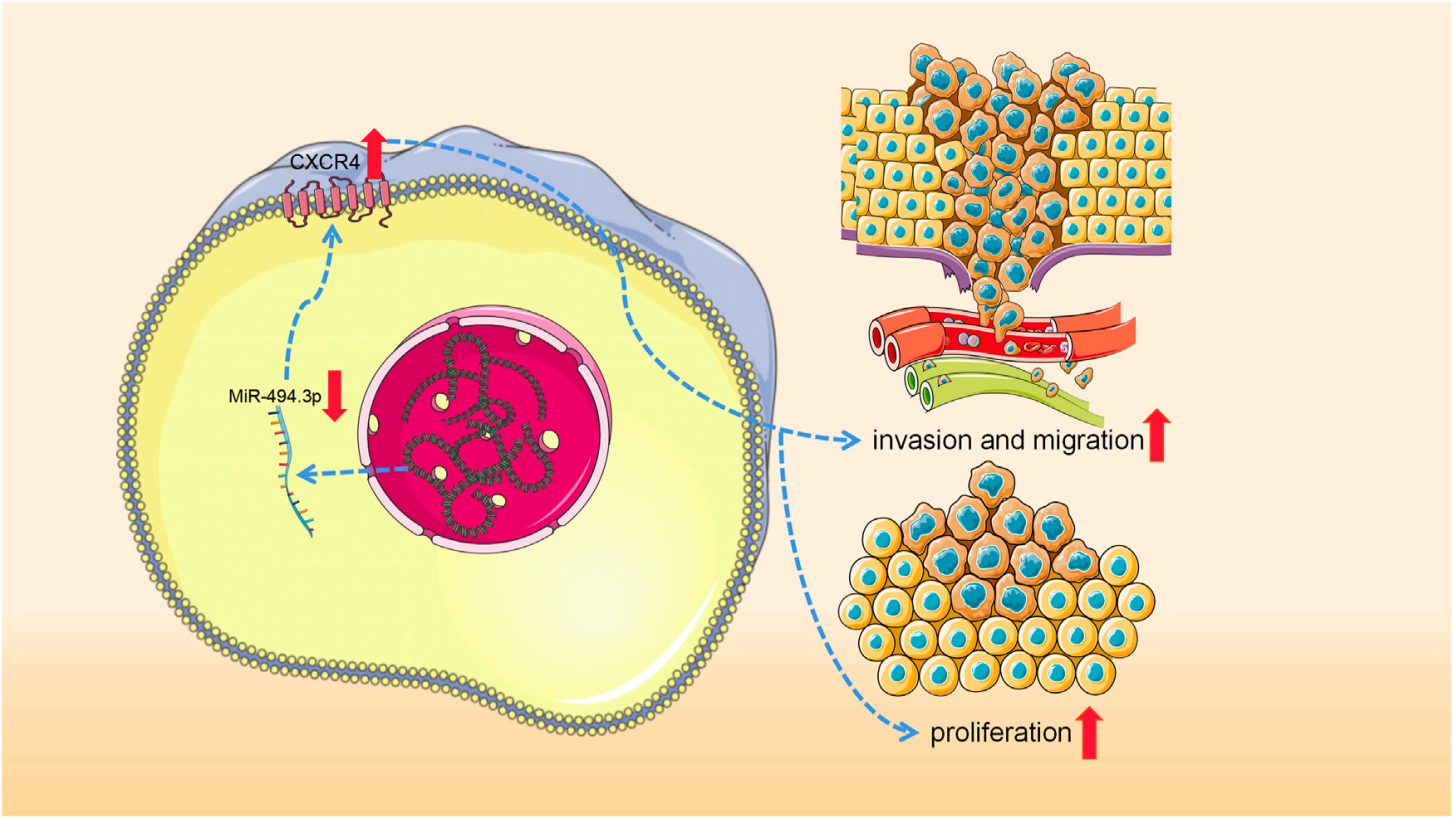
Low expression of miRNA-494-3p results in the increased expression of CXCR4, thus promoting the development of synovial sarcoma.

To sum up, CXCR4 can promote the incidence and progression of SS, and miR-494-3p plays a cancer suppressor role in SS through modulating CXCR4 (Figure 1).

### miR-17

miR-17 is located in the third intron on human chromosome 13, which is an amplified genomic site in many cancers (Yu et al., 2022). Moreover, many publications showed that miR-17 has been found in many human tissues. Research on miR-17 in the field of cancer has accumulated to some extent, and it has been reported that miR-17 is involved in the regulation of chordoma (Dong et al., 2021), bladder cancer (Geminder et al., 2001), myeloma (Xiang et al., 2021), nasopharyngeal carcinoma (Chen et al., 2016), and so on. In addition, previous research has indicated that miR-17, overexpressed miRNAs in cancers, may function as a carcinogenic substance and promote cancer development by negatively regulating tumor suppressor genes and/or genes that control cell differentiation or apoptosis (Zhang et al., 2007). In fact, miR-17 plays a carcinogenic role in these miRNAs mentioned above.

It is worth mentioning that there is a cyclin-dependent kinase inhibitor named p21 that inhibits cyclin-dependent kinases complex activity and can be considered to be a tumor suppressor (Abbas and Dutta, 2009). P21 could play a role in the regulation of miR-17 in some tumors, and miR-17 has been identified to directly target p21 in various cancers, according to some studies (Chen et al., 2016; Wang and Ji, 2018). For example, in chordoma tumors, in which miR-17 has a potential oncogenic role, the upregulation of miR-17 promoted cell proliferation, colony formation, and invasion *via* targeting p21 in chordoma cells (Dong et al., 2021). Furthermore, in bladder cancer, the sponging to miR-17 and the regulating of p21 expression are involved in the inhibition of circ-ITCH (a circular RNA) to bladder cancer progression (Yang et al., 2018). In addition, by targeting P21, miR-17 can promote the development of nasopharyngeal carcinoma (Chen et al., 2016).

In fact, there are also miR-17 and p21 in SS. Several researchers found that SS can generate oncoproteins, such as SS18-SSX1 and SS18-SSX2, which induces the expression of miR-17 (Minami et al., 2014). To identify the role of miR-17 in SS, they conducted a series of experiments whose results showed that miR-17 straightly targets the 3′-UTR of p21 mRNA and inhibits its expression. It is noteworthy that there is a broad-spectrum anticancer drug called doxorubicin, which can lead to the high expression of p21 and inhibit the development of SS. In their experiments, they found that p21 levels were drastically reduced in miR-17-overexpressing SS cells in a p53-independent manner and even in the presence of doxorubicin-induced giant p21 expression. Those resulted in the profound growth of SS in mice both *in vivo* and *in vitro*. Meanwhile, with the introduction of anti-miR-17 in these cells, the growth of these cells was decreased consistently with the rescued expression of p21. It is further verified that miR-17 acts in the development of SS through interaction with P21 (Minami et al., 2014).

In brief, SS cells generated SS18-SSX fusion oncoprotein when chromosomes 18 and X were ectopic. The expression of miR-17 is then promoted by these fusion oncoproteins. After then, miR-17 attaches to p21 mRNA and prevents it from performing its regular role. It is known that p21, as a cyclin-dependent kinase inhibitor, may block the G1/S transition of cells by targeting the Cyclin-CDK2 and Cyclin-CDK4 complexes and so fulfill its tumor suppressor role. When p21 interacts with miR-17, it becomes inactive, which implies that SS will continue to grow.

**FIGURE 2:**
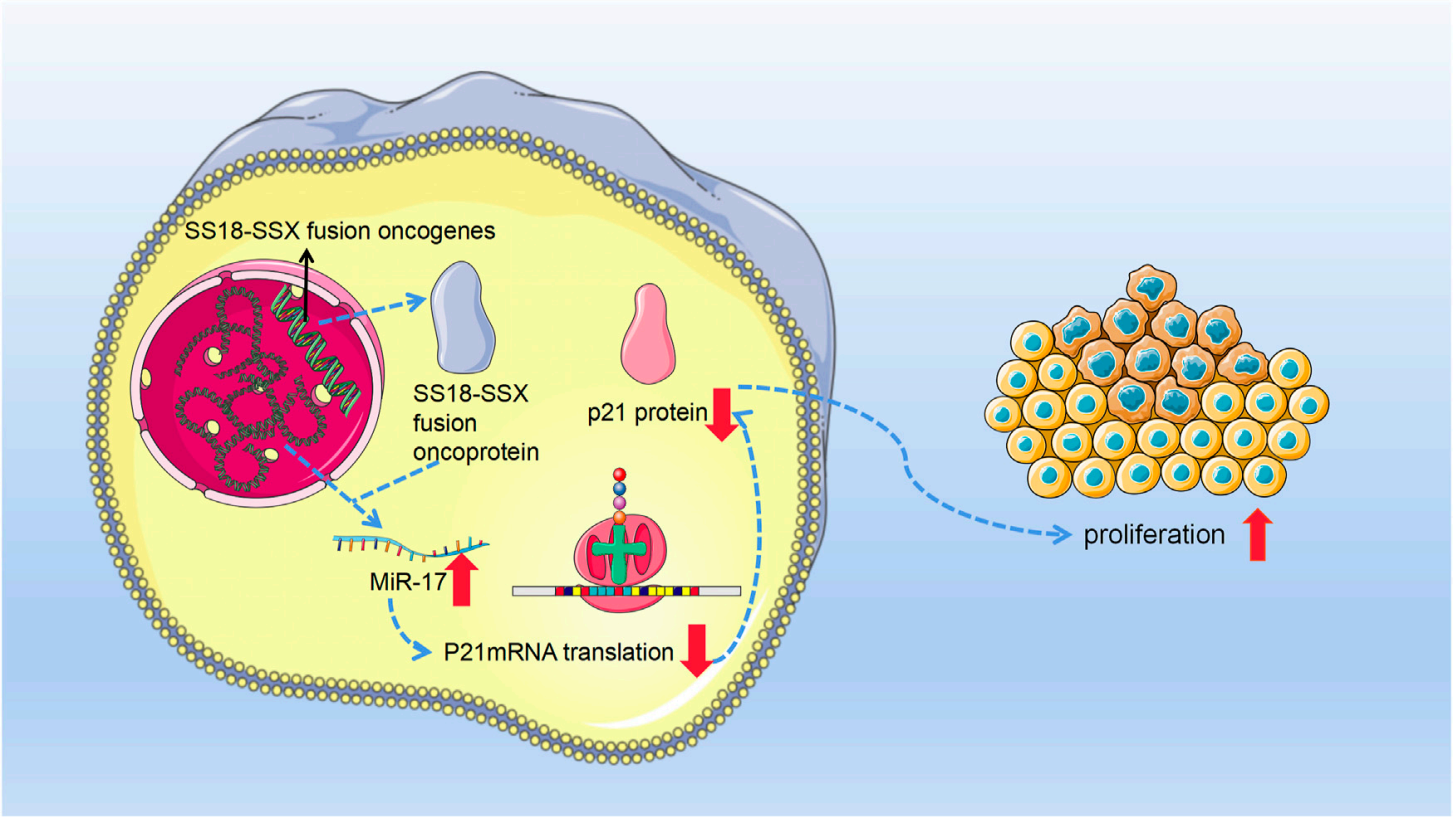
With the occurrence of chromosome 18 and X chromosome ectopic, synovial sarcoma cells produce synovial sarcoma (SS)18-SSX fusion oncoprotein. Then, these fusion oncoproteins promoted the expression of miR-17. Afterward, miR-17 binds to p21 mRNA and inhibits its normal function. Since in normal functioning, p21 can inhibit cell’s G1⁄S transition with Cyclin-CDK2 and Cyclin-CDK4 complexes as its targets and then fulfill its tumor suppressor role. Therefore, miR-17 finally leads to the invasive growth of synovial sarcoma cells by posttranscriptional suppression of p21.

In conclusion, miR-17 acts as an oncogene, causing SS cells to proliferate aggressively by directly suppressing the production of p21 (Figure 2).

### miR-214

MiR-214 lies in the antisense complementary sequence of the DNM3 gene, which is located in the q24.3 arm of human chromosome 1 NC_000001.11 (Weber, 2005; Scott et al., 2012). Several studies have reported that miR-214 presents in many tissues in humans and its upregulation and downregulation can cause a variety of different human malignancies, such as pancreatic (Zhang et al., 2010), cervical (Qiang et al., 2011), hepatoblastoma (Magrelli et al., 2009), hepatocellular (Duan et al., 2012), lung (Yanaihara et al., 2006), breast (Sempere et al., 2007), gastric (Yang et al., 2013), osteosarcoma (Wang et al., 2014), esophageal squamous cell carcinoma (Zhou and Hong, 2013), prostate (Srivastava et al., 20132013), ovarian (Yang et al., 2008), bladder (Ratert et al., 2013), and melanoma (Penna et al., 2011) cancers. Moreover, miR-214 can induce angiogenesis, target tumor suppressor genes, and serve as a potential tumor inhibitor, an anticancer therapeutic agent, and a biomarker (Sharma et al., 2015).

In fact, miR-214 also functions as a prognostic biomarker in SS. According to previous research, miR-214 functions in the development and progression of SS in cooperation with SS18-SSX1. As a character to SS which can be observed in most SS, SS18-SSX gene fusion proteins play a key role in the development of SS (Ladanyi, 2001).

Some researchers looked at the collaboration between miR-214 and SS18-SSX1 using a series of sarcoma induction assays and found that miR-214 and SS18-SSX1 were co-introduced into mice with SS. The co-introduction enhanced sarcoma onset, showing that miR-214 functions as a cooperative carcinogen in SS genesis. Based on the findings of Tanaka et al. (2020), miR-214 significantly accelerated the development of SS18-SSX1-induced SS.

*In vitro*, overexpression of miR-214 in SS cells did not improve cell growth, migration, or invasion capacities, indicating that it may promote tumor formation in a cell nonautonomous manner (Fang and Greten, 2011; Bellazzo et al., 2018). MiR-214 may promote intracellular signaling of inflammatory cells to release tumor growth-promoting substances. It was also found that cytokine Genes Cxcl15, which encodes IL-8 was upregulated in SS and induces migration and activation of granulocytes and macrophages (Baggiolini and Clark-Lewis, 1992; Xie, 2001). IL-8 expression was shown to be higher in a variety of human cancers, and it was linked to more aggressive phenotypes (Abdul-Aziz et al., 2017; Manfroi et al., 2017; Kahraman et al., 2019; Maynard et al., 2020). Some researchers supposed that IL-8 enhances tumor cell invasiveness by interacting with the tumor microenvironment (Tanaka et al., 2020). The functional regulation of tumor-associated neutrophils by IL-8 has also been characterized as a poor prognostic factor for malignancies (Manfroi et al., 2017; Nie et al., 2019), which might explain why miR-214 was linked to a poor prognosis.

Existing evidence suggests that SS18-SSX1 and miR-214 expression cooperate in the development and malignant characteristics of SS. In short, the overexpression of miR-214 promotes the development of SS via modulating cytokine gene expression and the tumor microenvironment while interacting with SS18-SSX1. Because of its link to a poorer prognosis in SS, miR-214 may be applied as a prognostic biomarker (Tanaka et al., 2020).

### miR-92b-3p

MiR-92b-3p is a multifunctional miRNA located on human chromosome 1, and it has been found in many human tissues and cancers. Some reports indicated that the expression of miR-92b-3p is increased in some cancers, such as colorectal (Gong et al., 2018), prostate (Wang et al., 2021), and cervical (Kurata and Lin, 2018). Among these cancers, miR-92b-3p has a potential role in the occurrence and metastasis of colorectal cancer (Gong et al., 2018), and its upregulation is closely related to distant metastasis, lymph node metastasis, and poor prognosis in prostate cancer patients (Wang et al., 2021). In addition, miR-92b-3p can act as a cancer suppressor in pancreatic cancer (Long et al., 2017), a potential dynamic biomarker to monitor chemoresistance in small cell lung cancer (Li et al., 2021), and a potential biomarker for screening hepatocellular carcinoma (Sorop et al., 2020). Moreover, miR-92b-3p is reported to appear in human SS and is associated with SS.

There is previous research about miR-92b-3p in SS. Several researchers have found that miR-92b-3p was the only one that is clearly secreted from SS cells and might be a biomarker for SS by studying the expression patterns of serum cell-free miRNAs and then analyzing four upregulated miRNAs with statistical significance (Uotani et al., 2017b). To evaluate whether miR-92b-3p can be a useful biomarker of SS, they used mice to conduct some experiments and found that serum miR-92b-3p levels significantly decreased after tumor resection, with relevant data with statistical significance, indicating that the serum miR-92b-3p levels can reflect the tumor burden in experimental mice. By analyzing the serum miR-92b-3p levels in a validation cohort of SS patients, age-matched patients with benign tumors, and healthy individuals, they obtained the data that the sensitivity and specificity of serum miR-92b-3p levels were respectively 81.8% and 63.6%, which means that miR-92b-3p also can reflect the tumor burden to SS patients. Afterward, they discovered that serum miR-92b-3p expression levels may be applied in clinical tumor monitoring by assessing the demographics and clinical features of patients and healthy persons in the validation cohort and investigating certain cases. In their experiment, the miR-92b-3p expression level is considerably higher in the culture medium of SS cells rather than of STS cells, indicating that serum miR-92b-3p is effective in distinguishing SS patients from other STS patients and reflecting tumor load in SS patients (Uotani et al., 2017b).

In conclusion, miR-17 acts as an oncogene, causing SS cells to proliferate aggressively via posttranscriptional suppression of p21.

### miR-9

MiR-9 has three separate forms in humans: miR-9-1, miR-9-2, and miR-9-3, and they are respectively transcribed from different independent genomic loci mapping to chromosomes 1q22 (miR-9-1), 5q14.3 (miR-9-2), and 15q26.1 (miR-9-3) (Roese-Koerner et al., 2016). miR-9 has been found in multiple tissues in the human body and has been associated with the occurrence, development, and metastasis of a variety of cancers, such as lung, gastric, bladder, breast, cervical, prostate, and glioblastoma. Relevant research showed that miR-9 can play carcinogenic and inhibitory roles in gastric cancer and lung cancer, may have a carcinogenic role in prostate cancer, and also play different roles in some other cancers, such as inhibiting the expression of genes related to cell growth, angiogenesis, and migration (Khafaei et al., 2019). In addition, according to previous studies, miR-9 is a good biomarker for evaluating the prognosis of various cancers (Xu et al., 2016).

In fact, it has been reported in a study that miR-9 is significantly upregulated in SSs (over 30 times) (Yu et al., 2016). Therefore, there might be some connection between miR-9 and SS.

In previous research, several researchers thought that miR-9 can promote the migration and invasion of SS cells by targeting E-cadherin (also known as CDH1), which involved a process, namely, the epithelial–mesenchymal transition (EMT) (Xu et al., 2019). EMT is a process in which polarized epithelia lose their polarity under certain physiological or pathological conditions and are converted into interstitial cells with mobility and the ability to move freely between cellular matrixes (Nistico et al., 2012), which is linked to tumor invasion and metastasis. CDH1 as one of the common marks of EMT can take part in epithelial adhesion and desmosomal junction, cell differentiation, maintenance of cell morphology, and regulation of intercellular adhesion (Onder et al., 2008; Zeisberg and Neilson, 2009; Nistico et al., 2012). CDH1 is an essential intercellular adhesion molecule that inhibits tumor metastasis (Petrova et al., 2016), and its absence is a key marker of EMT and a need for epithelial tumor cells to invade.

These researchers mentioned above found the changes between miR-9 and CDH1 are significantly negatively correlated with statistical significance, according to bioinformatic analysis. Then, after some experiments and analysis, they found out that miR-9 can induce EMT of human SS cells as well as cell migration and invasion via targeting CDH1 (Xu et al., 2019). Overexpression of miR-9 boosted human SS cell proliferation, knockdown of miR-9 decreased cell proliferation, and overexpression of CDH1 reversed the impact of miR-9 on cell proliferation, according to the findings of the colony formation experiment. The findings, when combined with data analysis using RT-qPCR and western blot, revealed that miR-9 may target CDH1 in the mouse model, hence inducing EMT and enhancing human SS carcinogenesis in mice through activating the mitogen-activated protein kinase/extracellular signal-reduced kinase (MAPK/ERK) and Wnt/β-catenin signaling pathways (Xu et al., 2019).

The simplified process of that signal pathways function in SS can be summarized as follows. Overexpression of miR-9 stimulated the MAPK/ERK and Wntβ-catenin signaling pathways. In the Wnt/β-catenin signaling system, inappropriately activated Wnt genes may suppress the phosphorylation of β-catenin by the GSK3/APC/Axin complex, resulting in reduced phosphorylation degradation of β-catenin. The accumulating β-catenin may then attach to CDH1 to create a complex, which may aid tumor cell invasion and metastasis. Previous studies confirmed that MAPK signaling pathways act in tumor EMT process. The overexpression of miR-9 can significantly promote the expression of ERK proteins and then activate the MAPK/ERK signaling pathway, which stimulates proliferation and inhibits apoptosis of human SS cells (Xu et al., 2019).

**FIGURE 3:**
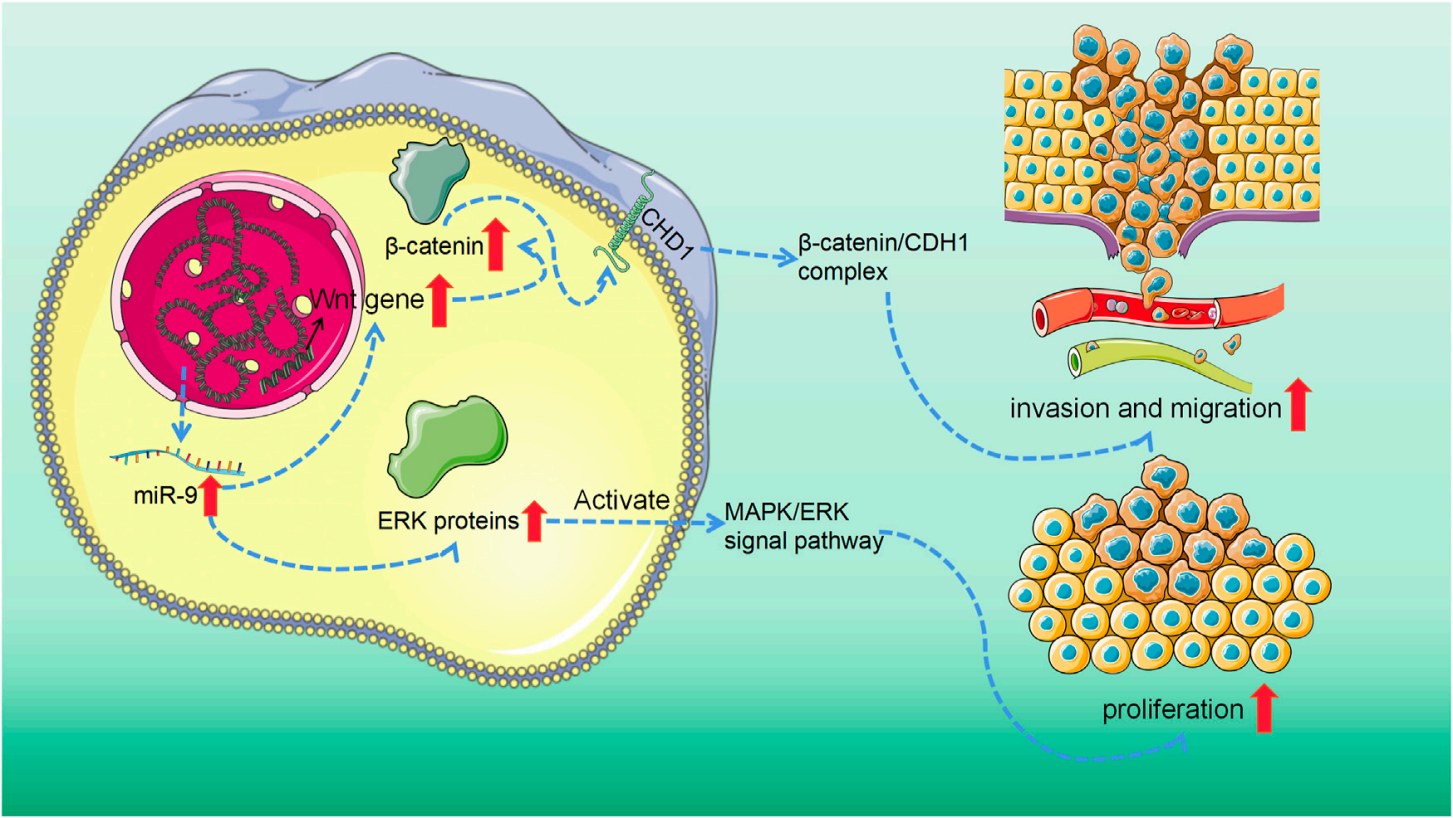
MiR-9 overexpression activated mitogen-activated protein kinase/extracellular signal-reduced kinase (MAPK/ERK) and Wnt/β-catenin signaling pathway. On the one hand, in the Wnt/β-catenin signaling system, the abnormally activated Wnt gene in tumor tissue can reduce the phosphorylation degradation of β-catenin. The accumulating β-catenin may then attach to CDH1 to create a complex, which may aid tumor cell invasion and metastasis. On the other hand, the overexpression of miR-9 can significantly promote the expression of ERK protein and then activate MAPK/ERK signaling pathway, to stimulate the proliferation of SS cells and inhibit apoptosis.

In other words, miR-9 promotes human SS by inducing EMT by targeting CDH1 and activating the MAPK/ERK and Wnt/β-catenin signal pathways (Figure 3).

## Clinical Significance of MicroRNA in Synovial Sarcoma

Various miRNAs play pivotal roles in SS from diagnosis and treatment to prognosis, and they even have a significant connection with the occurrence and development of SS. First, in the diagnosis aspect, there is a problem long time puzzled many medical workers who seek better ways to diagnose SS, which is the lack of useful biomarkers for SS. However, if there are more in-depth studies on miR-92b-3p, the problem may get well solved. In previous research, the related experiments in a validation cohort of SS patients, age-matched patients with benign tumors, and healthy individuals, successfully showed that miR-92b-3p could reflect the tumor burden of SS patients and could be a useful biomarker for SS, which gives us an inspiration that miR-92b-3p has a possibility to become a biomarker to SS in humans in the future (Uotani et al., 2017a).

As for the treatment of SS, considering that different miRNAs can function as tumor suppressors or oncogenes in SS, making full use of the role acted by miRNA to deal with SS is a feasible idea. For example, the difference in miR-494-3p between metastatic and nonmetastatic patients suggests that we may be able to use miRNAs to do something clinically relevant with enough studies (Pazzaglia et al., 2019).

Some miRNAs may have a role in SS prognosis. The experiment that the co-introduction of SS18-SSX and miR-214 into experimental mice led to the rapid development of SS and the finding that miR-214 could be the prognostic biomarker indicates that we can measure the level of miR-214 to preliminarily assess the prognosis of SS patients (Tanaka et al., 2020).

In fact, the clinical diagnostic value of miRNAs is important very much, and it is of great diagnostic value in many aspects of SS, such as diagnosis, treatment, and prognosis. If we can understand exactly how these miRNAs work in SS, we can not only take care of this disease as well as similar diseases and save many lives but also even apply the same thinking to other types of diseases in the future.

## Future Perspectives

It has previously shown that miRNA plays a role in the development of SS and may also provide new ideas for SS diagnosis and treatment. However, there are many obstacles to the current exploration of miRNA in SS.

First is that the mechanism by which miRNA plays a role in SS is unclear. The signal conduction pathway or specific mechanism between many miRNAs and SS is unclear, and most of the existing calculation methods that study miRNA regulation are based on a large number of miRNA and mRNA expressing data, using statistical tools to infer the relationship. However, it is important to pay attention to miRNA regulation in each cell, so a large amount of RNA sequencing expression data based on a large number of cells from a large number of cell groups may not cover the heterogeneity of miRNA regulated in a single cell in these people. With the current development and application of new technologies, single-cell RNA sequencing makes miRNA regulation at a single-cell level possible. Studies have been conducted using a hemi-cell genomics approach to generate single-cell miRNA mRNA co-sequencing expression data from 19 K562 hemi-cells and then apply Pearson correlation to identify miRNA targets (Wang et al., 2019). Based on this, the application of single-cell RNA sequencing technology to explore miRNA regulation in SS cells can be reasonably speculated. In addition, spatially resolved single-cell transcriptomics may be a new method for detecting miRNA regulation in SS cells. Spatially resolved single-cell transcriptomics can display the spatial heterogeneity distributed by miRNA in cells. The miRNA distribution in hepatocytes has been investigated by spatially resolved single-cell transcriptomics (Ben-Moshe et al., 2019). Likewise, the spatial distribution of miRNA in SS cells can be explored through spatially resolved single-cell transcriptomics. These emerging technologies provide new options for observing the mechanism of miRNA in SS, which is conducive to a deeper understanding and clarification of the mechanism of miRNA, and a clear direction for miRNA’s diagnosis and treatment in SS.

Secondly, to achieve the purpose of treatment, it is necessary to extract a specific miRNA and fed into patients to reach specific organs, tissues or cells. In the meantime, how to pass miRNA to specific targets more effectively must still be resolved. miRNA’s delivery includes viruses and nonvirus vectors, and the use of these systems has shortcomings. The limitations of the virus carrier are limited in large-scale production, immunogenicity, and toxic forces (Gardlik et al., 2005). Instead, the nonvirus vector overcomes the above problems in the virus carrier, but it has the disadvantage of low transfusion efficiency (Wang et al., 2016). According to available experiments, the disadvantages of one carrier can be compensated by the advantages of another carrier with synergy (Ban et al., 2019). Therefore, the use of a miRNA co-delivery vector may solve the problem of how to better pass miRNA to a specific target. These must be further explored in future clinical trials involving miRNA treatment.

Overall, in the future, through innovation and improvement of other related technologies, the application of miRNA in SS will be accelerated, which will provide new ideas for SS’s pathogenesis and treatment.

## Conclusion

Through extensive evidence and analysis, the important roles that certain miRNAs play in the pathogenesis and clinical application of SSs, such as occurrence, development, diagnosis, treatment, and prognosis, have been demonstrated. The five types of miRNAs described in the review have different connections with SS. First, for miR-494-3p, the modulation of CXCR4 mediated by the ectopic expression of miR-494-3p can suppress the proliferation and migration of the cells of SS, which means that miR-494-3p functioned as a tumor suppressor in SS. This implicates the possibility in clinical application that we can promote the expression of miR-494-3p or other related ways to inhibit the development of SS. Next, miR-17 can play a carcinogenic role and cause an aggressive growth of SS cells via posttranscriptional suppression of p21. Likewise, miR-9 can cause SS. It promotes human SS cancer by inducing EMT by targeting CDH1 and activating the MAPK/ERK and Wnt/β-catenin signaling pathways. Therefore, it may be of clinical significance to prevent and treat SS by inhibiting the effects of these two miRNAs. Because liquid biopsy based on serum miR-92b-3p expression levels may provide a unique strategy for monitoring tumor dynamics in SS, miR-92b-3p may be employed in the diagnosis of SS. Thus, miR-214 could be used as a prognostic biomarker because of its association with a worse prognosis of SS. Moreover, in collaboration with SS18-SSX1, the overexpression of miR-214 promotes the development of SS via modulating cytokine gene expression and the tumor microenvironment. As our understanding of miRNAs grows, we can provide more new ideas for the prevention and treatment of SSs and other similar diseases.
